# Using cortical auditory-evoked potential responses to verify the objective benefit of aural rehabilitation in adult cochlear implant users

**DOI:** 10.3389/fnins.2026.1815357

**Published:** 2026-07-22

**Authors:** Caris Bogdanov, Wilhelmina H. A. M. Mulders, Helen Goulios, Welber Marinovic, Dayse Tavora-Vieira

**Affiliations:** 1 School of Human Sciences, University of Western Australia, Perth, WA, Australia; 2 Department of Audiology, Fiona Stanley Fremantle Hospitals Group, Perth, WA, Australia; 3 Faculty of Health Sciences, Curtin School of Population Health, Curtin University, Perth, WA, Australia; 4 Division of Surgery, Medical School, University of Western Australia, Perth, WA, Australia; 5 Faculty of Health Sciences, Curtin School of Allied Health, Curtin University, Perth, WA, Australia

**Keywords:** auditory training, aural rehabilitation, cochlear implant, cortical auditory-evoked potentials, speech perception

## Abstract

**Introduction:**

Cortical auditory-evoked potential (CAEP) responses are used clinically to assess auditory processing and support cochlear implant (CI) mapping procedures post-implantation. This study aims to examine CAEP responses as an objective measure of neurophysiological changes following a structured Aural Rehabilitation Program (ARP) in long-term adult CI users, with poor baseline speech perception performance. Advancing our understanding of the mechanisms underlying improved post-implantation outcomes in poor performing CI users may inform the development of targeted interventions and refinement of evidence-based rehabilitation strategies.

**Methods:**

Adult unilateral CI users with post-lingual deafness were recruited for an intensive clinician-directed ARP incorporating structured active listening exercises. The ARP consisted of weekly 1-h face-to-face sessions over 10 weeks with repeated assessments at baseline (T0) and 10-week intervals (T10, T20, T30). Participants were allocated to immediate (Group 1), 10-week delay (Group 2), or 20-week delay (Group 3) ARP groups, while a control group completed assessments without intensive ARP. Speech perception in quiet [CNC phoneme and word scores (%)] and electrically-evoked CAEP (eCAEP) response [P1-N1-P2 peak latencies (ms) and amplitudes (μV)] measures were collected at each time point for all groups. The repeated measures design incorporated staggered pre- and post-ARP periods for Groups 1–3 (T0-T10, T10-T20, and T20-T30, respectively). CNC and eCAEP measures were analyzed pre- and post-ARP intervention and across assessment intervals (T0-T30) to evaluate rehabilitation effects and account for natural variability and potential ongoing adaptation in auditory performance over time, independent of ARP-related changes.

**Results:**

No significant changes were observed in eCAEP response latencies or amplitudes from pre- to post-rehabilitation. However, the ARP intervention group demonstrated significantly higher CNC phoneme and word recognition scores compared to the control group in both implant-alone and binaural listening conditions post-rehabilitation.

**Discussion:**

No measurable neurophysiological changes were observed in eCAEP responses from pre- to post-rehabilitation. However, the ARP intervention group demonstrated behavioral improvements, reflected in marginal increases in speech perception scores at both phoneme and word recognition levels, which were not observed in the control group. These findings highlight the clinical value of structured aural rehabilitation in supporting improved outcomes for long-term adult CI users with poor baseline performance.

## 1 Introduction

A cochlear implant (CI) is an auditory prosthesis that restores sound perception in individuals with severe-to-profound hearing loss and poor speech discrimination unresolved by hearing aids, bypassing damaged cochlear structures to directly stimulate the auditory nerve (Naples and Ruckenstein, 2020). Studies demonstrate consistent speech recognition and quality of life improvements following cochlear implantation, though outcomes vary widely among recipients (Ma et al., 2023; Moberly et al., 2016). Despite advancements in device design, surgical techniques, and programming, post-CI speech recognition performance remains highly variable (Holden et al., 2013; Ma et al., 2023; Moberly et al., 2016; Zhao et al., 2020). While many CI users demonstrate significant improvement, some are classified as *poor performers* with minimal or no gains achieved (Boisvert et al., 2020; Lenarz et al., 2012; Völter et al., 2022b). Speech perception improvement occurs primarily within the first 3 months post-implantation (Ma et al., 2023), and typically stabilizes by 18–24 months post-implantation (Moberly et al., 2018). However, many users report dissatisfaction, particularly in complex auditory environments, where speech recognition benefits are markedly reduced compared to quiet conditions (Ma et al., 2023). Standard clinical evaluations for poor performance rely primarily on medical investigations, including computed tomography (CT) to assess electrode positioning and hardware integrity checks. However, these measures often do not identify issues warranting revision surgery (Harris et al., 2016). While Audiologists employ a range of programming and fitting strategies to improve performance, audiological programming alone is insufficient to fully address the variability in speech recognition outcomes (Martins and Goffi-Gomez, 2021; Távora-Vieira and Marino, 2019).

Given that the primary goal of cochlear implantation is to restore access to sound, facilitating both environmental awareness and speech perception to support communication in daily life (Bierbaum et al., 2020; Ebrahimi-Madiseh et al., 2020), users with limited early post-implantation speech recognition gains may require additional support to maximize their auditory potential. These individuals may benefit from interventions such as the implementation of communication strategies, speech-language pathologist-led rehabilitation and computer-based auditory training, all of which have been shown to enhance device adaptation and in turn, improve speech recognition outcomes (Bernstein et al., 2021; Dornhoffer et al., 2024a; Dornhoffer et al., 2024b; Henshaw and Ferguson, 2013; Moberly et al., 2018; Schumann et al., 2015).

The rehabilitation of hearing with a CI relies on central auditory system plasticity, enabling adaptation to the new and unnatural electrical input provided by the implant (Glennon et al., 2020; Moore and Shannon, 2009). Aural rehabilitation, encompassing counseling, instruction, and auditory training, plays a crucial role in enhancing speech perception, auditory processing, and communication strategies, particularly for CI users adapting to new auditory input (Bernstein et al., 2021; Boothroyd, 2007; Dornhoffer et al., 2021; Sweetow and Palmer, 2005). Post-implantation, patients must learn to interpret the electrically coded speech signal, a process that occurs naturally for some, but for most CI users requires intentional practice and targeted auditory training (Fu and Galvin, 2007; Fu et al., 2004). Auditory training is typically categorized into patient-directed, at-home exercises and clinician-directed sessions, incorporating exercises that encompass both passive and active listening activities (Boothroyd, 2010; Dornhoffer et al., 2022; Henshaw and Ferguson, 2013; Olson, 2015). Passive exercises include listening to recorded speech, audiobooks, radio, or television, allowing patients to familiarize themselves with speech sounds in various contexts. Active exercises involve more structured activities, such as interactive communication with feedback from partners, speech-tracking tasks, and computer-based auditory training programs (Dornhoffer et al., 2024b; Henshaw and Ferguson, 2013; Olson, 2015; Reis et al., 2021; Schumann et al., 2015). Overall, these exercises are designed to improve listening effort, speech recognition, and overall quality of life by promoting auditory processing skills (Dornhoffer et al., 2024b).

Despite the development of numerous auditory training exercises, no standardized protocols have been established for use in the adult CI population, and evidence supporting the efficacy of specific interventions remains limited (Cambridge et al., 2022; Dornhoffer et al., 2024a). As a result, audiologists face challenges in recommending targeted rehabilitation strategies, relying on empirical approaches rather than evidence-based guidelines (Dornhoffer et al., 2024a; Harris et al., 2016; Sweetow and Palmer, 2005). The lack of robust evidence on the effectiveness of specific training resources limits the ability to optimize rehabilitation for CI users, highlighting the need for further research to identify and validate the benefits of auditory training interventions.

Performance outcomes in CI users are commonly evaluated through speech perception tests, including the Consonant-Nucleus-Consonant (CNC) word test (Gifford et al., 2008; Peterson and Lehiste, 1962) and Bamford-Kowal-Bench (BKB) sentence test (Bench et al., 1979; Gifford et al., 2008), as well as quality-of-life (QoL) questionnaires, such as the Speech, Spatial, and Qualities of Hearing Scale (SSQ) (Gatehouse and Noble, 2004). However, measuring the benefits of aural rehabilitation using traditional speech materials presents a significant challenge (Moberly et al., 2025), as it is difficult to differentiate between genuine rehabilitation-induced improvements and learning effects over time (Boothroyd et al., 1985; Henshaw and Ferguson, 2013). Repeated exposure to the same test materials can lead to procedural learning, test-retest improvements, and lexical familiarity, confounding true rehabilitative gains (Parmar et al., 2022; Yund and Woods, 2010). This issue is particularly relevant in CI users, where perceptual learning can enhance speech recognition independently of rehabilitation (Henshaw and Ferguson, 2013; Yund and Woods, 2010). To address these limitations, electrophysiological measures, such as cortical auditory evoked potential (CAEP) responses, offer an objective method to assess auditory processing changes beyond learned test responses (Anderson and Kraus, 2013; Kelly et al., 2005; Martin et al., 2008). These approaches may provide a more accurate representation of aural rehabilitation benefits while minimizing the confounding effects of test familiarity and repetition.

Research has demonstrated the utility of CAEPs in evaluating the integrity of the auditory system and tracking cortical maturation in CI users by assessing processing along the auditory pathway to the cortex (Martin et al., 2008). Furthermore, studies have explored the relationship between specific CAEP components and outcomes post-CI, demonstrating evidence of enhanced N1 amplitude and decreased latency with improved auditory discrimination (Sandmann et al., 2015), and prolonged P2 latency in poorer CI performers (Kelly et al., 2005). In addition, the use of CAEP measures has been validated as an objective tool for verifying and optimizing CI mapping, contributing to improved speech recognition outcomes (Távora-Vieira et al., 2018; Távora-Vieira et al., 2022a; Távora-Vieira et al., 2022b), irrespective of specific patient or device factors (Bogdanov et al., 2023). Recent studies indicate that increased device usage enhances electrically-evoked CAEP (eCAEP) responses and improves waveform morphology, demonstrating cortical adaptation over time (Bogdanov et al., 2024). Given the growing body of literature supporting the use of CAEPs as a clinical tool for monitoring patient-specific auditory changes and optimizing performance outcomes, a key question remains concerning whether CAEPs can be used as a measure of aural rehabilitation benefit rather than traditional speech testing.

Examining the effects of auditory training on CAEP measures may provide an objective method to evaluate neurophysiological changes following aural rehabilitation and the mechanisms underlying improved outcomes in adult CI users. Advancing our understanding could help synthesize the benefits of rehabilitation to address current limitations in post-implantation care by developing targeted interventions and refining evidence-based auditory training strategies to improve rehabilitation practices. Ultimately, these advancements have the potential to enhance performance, lead to more consistent outcomes, and reduce variability among CI users. Therefore, the present study aims to investigate the use of CAEP measures as an objective method to assess neuroplastic changes following aural rehabilitation in adult CI users. The primary objective is to evaluate whether structured auditory training improves speech perception in long-term CI users with poor baseline performance, and to examine if these behavioral improvements are reflected in measurable changes in CAEP responses.

## 2 Materials and methods

### 2.1 Ethics

This study was designed and conducted in accordance with the Declaration of Helsinki, and ethics approval was obtained from the South Metropolitan Area Health Service, Human Research Ethics Committee (Reference Number: 3258). Written informed consent was obtained from all participants.

### 2.2 Participants

Participants were recruited from the audiology department at a tertiary hospital in Western Australia. Participants included in the study were adult (18–90 years) unilateral CI users with acquired post-lingual bilateral or single-sided deafness. Eligibility required ≥ 12 months of CI experience and Consonant-Nucleus-Consonant (CNC) test (Peterson and Lehiste, 1962) phoneme scores (implant-alone) within the lowest performance percentile ( < 60%) among CI patients at the implant center. Patients with phoneme scores > 60% (implant-alone) and those unable to comply with weekly rehabilitation sessions were excluded.

### 2.3 Cognition screening

Participant cognitive function was assessed using the Hearing-Impaired Montreal Cognitive Assessment (Hi-MoCA) screening, administered pre-enrolment in the ARP by a certified clinician. This tool was selected for its quick, easy administration and reliable screening of cognitive impairment in individuals with severe hearing loss (Lin et al., 2017).

### 2.4 eCAEP response recording

Participant eCAEP responses were elicited by direct electrical stimulation using the MAESTRO 9.0.1 (MED-EL, Innsbruck, Austria) eABR module and recorded via the Bio-logic Navigator Pro (Natus, Pleasanton, CA). Signal synchronization was achieved using a trigger cable connecting the CI programming interface and the Bio-Logic recording equipment. The eABR module presented the electrical burst stimuli at a rate of 0.9 Hz and with a duration of 70 ms. Stimuli were composed of a series of biphasic symmetric alternating pulses, each with a phase duration of 40 μs, presented at a stimulation rate of 1 kHz. Responses were recorded from 60 ms pre-stimulus to 500 ms post-stimulus onset, with at least 100 epochs per stimulus to ensure the exclusion of electrical artifacts generated by the CI. To obtain eCAEP responses, stimuli were presented at participant MCL levels for three electrode contact positions: electrode 1 (apical), 6 (medial), and 11 (basal). The eCAEP responses were recorded non-invasively using a three-electrode montage, consisting of a non-inverting, inverting, and ground electrode, placed on the vertex (Cz), mastoid contralateral to the CI, and forehead, respectively. Electrode impedance was maintained below 5 kΩ and the impedance differences between the electrodes were maintained at < 2 kΩ. Participant responses were recorded until 100 valid averages were obtained. The signal was filtered using an analog bandpass filter (0.3–100 Hz), with a 60 Hz notch filter applied if required. Participants were instructed to stay relaxed and minimize muscle contraction during the recording session. Alertness was monitored by the supervising clinician. Data reliability was confirmed by evaluating the reproducibility of CAEP recordings across repeated measurements.

### 2.5 P1-N1-P2 complex

The eCAEP data were exported in txt format for further analysis using Python (Python Software Foundation, version 3.9). To validate the accurate identification of present and absent eCAEP responses, two experienced audiologists independently visually inspected the waveform morphology of the P1, N1, and P2 components. The eCAEP responses were deemed present only when both audiologists agreed on the presence of the P1-N1-P2 complex. Response amplitudes (μV) and latencies (ms) were measured for P1, N1, and P2 components.

### 2.6 Speech perception testing

Speech perception in quiet was assessed using phoneme and word scores (%) obtained by the CNC word test (Peterson and Lehiste, 1962). Speech perception testing was conducted under two listening conditions in line with the standard clinical protocol. Participants were tested in an implant-alone condition using only the CI with the contralateral ear occluded, and in a binaural condition using the CI in combination with the contralateral ear, with hearing aid amplification provided when available and clinically indicated. All testing was performed in an anechoic free-field environment using a loudspeaker placed at 0° azimuth angle, one meter from the seated participant.

### 2.7 Aural rehabilitation program

The Aural Rehabilitation Program (ARP) was delivered over a 10-week intensive period, with participants attending 1-h, clinician-directed sessions once weekly. The intervention consisted of structured, active listening exercises using the MED-EL Adult Rehabilitation Kits (MED-EL, 2020), a standardized, freely accessible resource designed to support widespread implementation of auditory training for CI users. This resource comprises 13 kits that incorporate targeted auditory training activities, including speech perception tasks, phoneme discrimination, word recognition, and sentence-level listening exercises. A qualified clinician guided participants through one kit per session, with progression through the program following a sequential order across the 10 weeks. Task difficulty was individually tailored to participants’ progress and adjusted as required, including the provision or removal of visual cues (e.g., lip reading) and modifications to listening conditions, such as the introduction of background noise. Participants were encouraged to supplement clinician-led sessions with self-directed home-based training. This included active exercises using rehabilitation applications and passive listening activities, such as exposure to recorded speech (i.e. audiobooks, radio broadcasts, and television programs).

### 2.8 Data collection protocol

**FIGURE 1:**
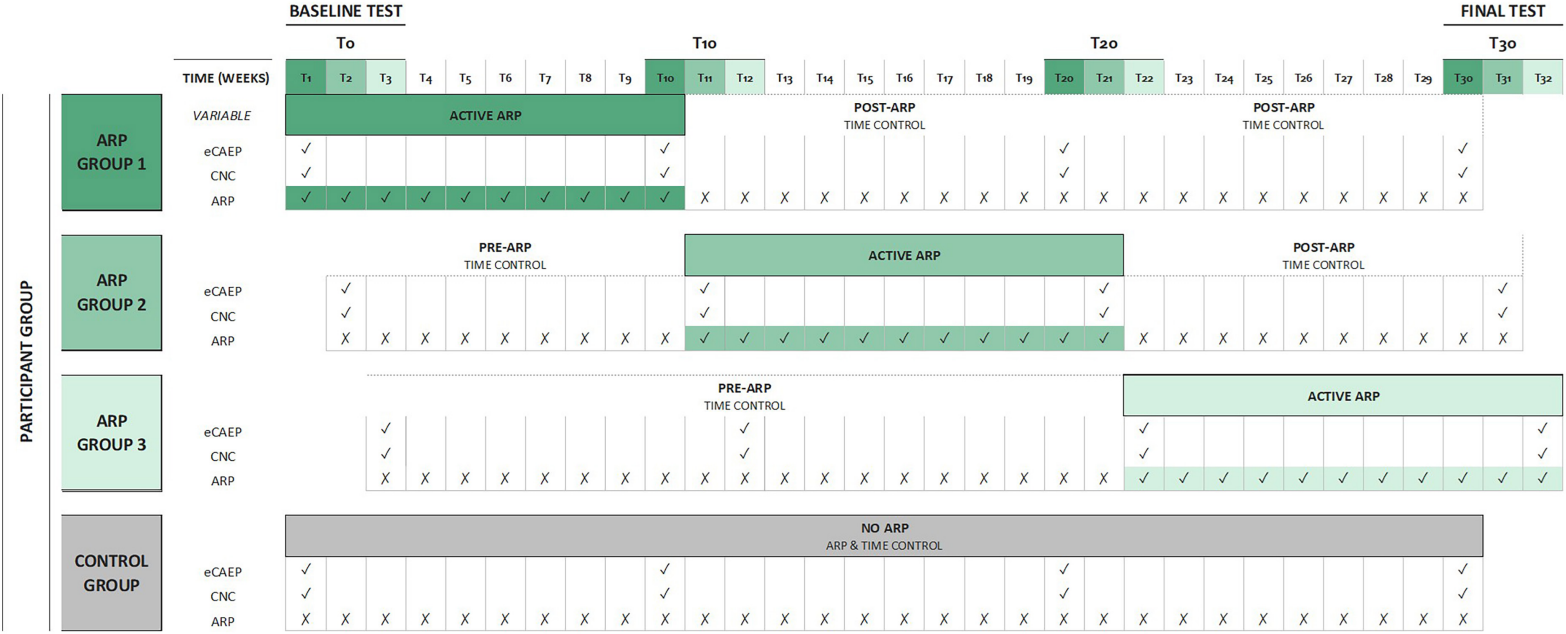
Aural rehabilitation program (ARP) and data collection timeline illustrating the 30-week repeated-measures design, from baseline (T0) to each 10-week assessment interval (T10, T20, and T30). The design incorporates a 10-week intensive ARP delivered in a staggered sequence across groups. Shaded rows with tick symbols (✓) indicate the active 10-week ARP intervention period for each group, while cross symbols (×) indicate non-intervention (control) periods. Pre- and post-ARP assessments were conducted at T0-T10 (Group 1), T10-T20 (Group 2), and T20-T30 (Group 3), respectively. Tick symbols (✓) indicate data collection time points for both electrically evoked CAEP (eCAEP) responses [latencies (ms) and amplitudes (μV)] and speech perception in quiet [CNC phoneme and word scores (%)].

The ARP consisted of weekly 1-h face-to-face sessions over 10 weeks. Participants completed the ARP within a 30-week repeated-measures design, with assessments conducted at baseline (T0) and at 10-week intervals (T10, T20, T30). This allowed intra-patient comparisons pre- and post-rehabilitation as well as across assessment intervals (T0-T30), to account for natural variability and potential ongoing adaptation in auditory performance, independent of the ARP intervention. Eligible patients were informed about the program and invited to participate. Following comprehensive counseling and providing informed consent, participants voluntarily enrolled and were assigned program start dates using a quasi-experimental design based on patient availability. Participants were assigned to one of three ARP groups: immediate commencement (ARP Group 1), 10-week delay (ARP Group 2), or 20-week delay (ARP Group 3). The repeated-measures design incorporated staggered intervention periods across groups, with pre- and post-ARP assessments occurring at T0-T10 (Group 1), T10-T20 (Group 2), and T20-T30 (Group 3), respectively (Figure 1). Control group participants declined intensive weekly ARP, however, completed outcome measure testing at all-time points (T0-T30). Participants with excessive absences or inconsistent weekly attendance due to illness or personal reasons were excluded from the analysis.

### 2.9 Statistical analysis

All statistical analyses were conducted using R statistics and R Studio software (Team, 2025). Normality (via the Shapiro-Wilk test) and equal variance (via the F test) were determined on the basis of which parametric or non-parametric data analysis was conducted. The Mann-Whitney U test was used to compare quantitative variables between two independent groups stratified by categorical variables. For comparisons involving more than two groups, the Kruskal-Wallis H test was conducted. To control for the risk of type 1 error, we implemented the Holm method to adjust the significance level for multiple comparisons. Linear mixed model (LMM) analysis using the “lmer” function from the “lme4” package (Version 2.0-1; Bates et al., 2015) was performed to analyse the effect of time (baseline, T10, T20, T30), ARP sub-group (Group 1, Group 2, Group 3), and test electrode contact position (apical, medial, basal) on eCAEP response amplitude (μV) and latency (ms) for each P1, N1, and P2 waveform component. Random intercepts for participants (ID) were included to account for individual variability. Follow-up pairwise comparisons were conducted using Type III Analysis of Variance (ANOVA) with the “emmeans” function [Version 2.0.3; (Searle et al., 1980, Lenth et al., 2019)]. Estimated marginal means were extracted for plots to generate summary values projected from the LMM, to account for variability across trial observations and random effects, providing a representation of the statistical analysis that was more robust than raw means.

## 3 Results

### 3.1 Participants

A total of 35 adult long-term CI users (18 males, 17 females) participated in this study. All participants were implanted under the same surgical team with MED-EL electrode arrays: Standard, FORM24, FLEX24, FLEX26 or FLEX28, and received one of the following speech processors: SONNET, SONNET 2 or RONDO 3 (MED-EL, Innsbruck, Austria). Participant device characteristics are summarized in Table 1.

**TABLE 1:** Summary of participant demographics, hearing loss onset (nature and etiology), device information and duration of experience at time of enrolment.

Demographic data				Hearing loss onset		Device information					Device experience	
ARP group	Gender	Age at implantation (Yrs)	Age at enrolment (Yrs)	Nature	Etiology	Implant ear	Implant generation	Electrode array	Audio processor	STIM mode	Years	Months
Group 1	M	77	79	Sudden	ISSNHL	Left	SYNCHRONY 2	FLEX28	SONNET 2	BIPHASIC	1	10
Group 1	M	84	86	Gradual	NIHL	Left	SYNCHRONY 2	STANDARD	SONNET 2	BIPHASIC	2	11
Group 3	F	67	82	Gradual	Rubella	Left	SONATA	STANDARD	SONNET 2	BIPHASIC	15	3
Group 3	M	72	74	Gradual	ME Pathology	Left	SYNCHRONY 2	FLEX26	SONNET 2	BIPHASIC	1	9
Group 3	M	78	81	Gradual	Unknown	Left	SYNCHRONY 2	FLEX28	SONNET 2	BIPHASIC	2	10
Group 1	M	80	83	Gradual	NIHL	Left	SYNCHRONY 2	FLEX24	RONDO 3	BIPHASIC	2	4
Group 2	F	75	80	Gradual	Ménière’s Disease	Right	SYNCHRONY 2	FLEX28	SONNET 2	BIPHASIC	4	9
Group 1	M	72	77	Sudden	Vascular	Left	SYNCHRONY 2	FLEX28	SONNET 2	BIPHASIC	4	10
Group 2	M	66	75	Gradual	Ménière’s Disease	Left	SYNCHRONY 2	FLEX28	SONNET	BIPHASIC	9	0
Group 1	M	82	87	Gradual	Otosclerosis	Right	SYNCHRONY 2	FLEX28	SONNET 2	BIPHASIC	4	9
Group 3	F	76	84	Gradual	Presbycusis	Left	SYNCHRONY 2	FLEX28	SONNET 2	BIPHASIC	7	7
Group 2	F	52	56	Sudden	Trauma	Right	SYNCHRONY 2	FLEX28	SONNET 2	BIPHASIC	3	7
Group 2	F	82	85	Gradual	Vascular	Right	SYNCHRONY 2	FLEX28	SONNET 2	BIPHASIC	2	11
Group 2	F	40	44	Sudden	ME Pathology	Left	SYNCHRONY 2	FLEX28	SONNET 2	TRIPHASIC	3	4
Group 3	F	64	68	Sudden	ME Pathology	Right	SYNCHRONY 2	FLEX28	SONNET 2	BIPHASIC	8	0
Group 2	F	62	63	Gradual	Ménière’s Disease	Left	SYNCHRONY 2	FLEX28	SONNET 2	TRIPHASIC	1	6
Group 1	F	71	74	Sudden	Otosclerosis	Right	SYNCHRONY 2	FLEX28	SONNET 2	BIPHASIC	3	9
Group 1	F	76	81	Gradual	Presbycusis	Right	SYNCHRONY 2	FLEX28	SONNET 2	BIPHASIC	5	8
Group 2	F	62	64	Gradual	ME Pathology	Right	SYNCHRONY 2	FLEX28	SONNET 2	BIPHASIC	2	0
Group 2	F	76	78	Sudden	Virus	Left	SYNCHRONY 2	FLEX28	SONNET 2	BIPHASIC	2	0
Group 3	F	69	77	Gradual	Presbycusis	Right	SYNCHRONY 2	FLEX28	SONNET	BIPHASIC	8	5
Group 3	F	77	82	Sudden	ISSNHL	Right	SYNCHRONY 2	FLEX24	SONNET	BIPHASIC	5	8
Group 1	F	80	81	Gradual	Presbycusis	Right	SYNCHRONY 2	FLEX28	SONNET 2	BIPHASIC	1	7
Group 3	M	74	82	Sudden	ISSNHL	Right	SYNCHRONY 2	FLEX28	SONNET	BIPHASIC	8	6
Control	M	86	89	Gradual	Ménière’s disease	Right	SYNCHRONY 2	FLEX28	SONNET 2	BIPHASIC	1	0
Control	M	75	78	Sudden	Unknown	Left	SYNCHRONY 2	FLEX26	SONNET 2	BIPHASIC	2	4
Control	F	87	91	Sudden	Unknown	Right	SYNCHRONY 2	FLEX28	SONNET 2	BIPHASIC	1	0
Control	M	81	83	Gradual	Otosclerosis	Left	SYNCHRONY 2	FLEX28	SONNET 2	BIPHASIC	1	9
Control	M	11	22	Gradual	Genetic	Left	SYNCHRONY	FLEX24	RONDO 3	BIPHASIC	9	5
Control	M	82	86	Gradual	Presbycusis	Right	SYNCHRONY 2	FLEX28	RONDO 3	BIPHASIC	3	3
Control	M	80	84	Gradual	NIHL	Right	SYNCHRONY 2	FLEX28	SONNET 2	BIPHASIC	4	0
Control	F	83	84	Gradual	Ototoxicity	Left	SYNCHRONY 2	FLEX28	SONNET 2	BIPHASIC	1	1
Control	M	75	77	Sudden	Trauma	Right	SYNCHRONY 2	FORM24	SONNET	BIPHASIC	1	1
Control	M	68	72	Gradual	Otosclerosis	Left	SYNCHRONY 2	FLEX28	SONNET 2	BIPHASIC	1	4
Control	M	88	91	Gradual	Presbycusis	Right	SYNCHRONY 2	FLEX28	SONNET 2	BIPHASIC	1	8

ISSNHL, Idiopathic Sudden Sensorineural Hearing Loss; NIHL, Noise-Induced Hearing Loss; ME, Middle Ear.

### 3.2 Participant groups

A total of 24 participants completed the ARP, divided into three groups based on program commencement: immediate (T0; Group 1), 10-week delay (T10; Group 2), and 20-week delay (T20; Group 3). The median age at enrolment for each participant group was 81 years (range 74–87 years), 77 years (range 56–85 years), and 79 years (range 44–84 years) for the T0, T10 and T20 groups, respectively. The control group consisted of 11 participants with a median age at enrolment of 84 years (range 22–91 years). Group comparisons were conducted using the Kruskal-Wallis H test, which showed no significant difference in age at enrolment [χ^2^(3) = 4.96, *p* = 0.174] among groups. A significant difference was observed for age at implantation [χ^2^(3) = 8.85, *p* = 0.031]; however, *post-hoc* Dunn’s tests with Holm correction revealed no significant pairwise differences after multiple comparison adjustment across participant groups. Table 2 presents the individual adjusted *p*-values for the post hoc analysis of implant age across groups.

**TABLE 2:** Participant age at implantation by group, with Kruskal-Wallis rank sum test and Dunn’s *post hoc* analysis between groups.

Age at implant (years)	Comparison		*Z*-value	*p*-value	*p*-value (adj)
Group (*n*) Median (IQR)	ARP group 1 (8) 78.5 (5.5)	ARP group 2 (8) 70.5 (14.2)	1.72	0.085	0.255
Group (*n*) Median (IQR)	ARP group 1 (8) 78.5 (5.5)	ARP group 3 (8) 70.5 (8.25)	1.97	0.049	0.197
Group (*n*) Median (IQR)	ARP group 2 (8) 70.5 (14.2)	ARP group 3 (8) 70.5 (8.25)	0.24	0.807	0.807
Group (*n*) Median (IQR)	ARP group 1 (8) 78.5 (5.5)	Control group (11) 81 (9.5)	−0.29	0.769	1
Group (*n*) Median (IQR)	ARP group 2 (8) 70.5 (14.2)	Control group (11) 81 (9.5)	−2.15	0.032	0.159
Group (*n*) Median (IQR)	ARP group 3 (8) 70.5 (8.25)	Control group (11) 81 (9.5)	−2.41	0.016	0.096

### 3.3 Total device experience

The total duration of CI experience was captured at the time of enrolment for baseline testing. Median CI experience was highest in ARP Group 3 (7.85 years, *IQR*: 5.45, *n* = 8), followed by ARP Group 1 (3.15 years, *IQR*: 2.29, *n* = 8) and ARP Group 2 (2.90 years, *IQR*: 3.12, *n* = 8), with the control group demonstrating the lowest CI experience (1.80 years, *IQR*: 1.75, *n* = 11). A Kruskal-Wallis test revealed a significant difference in CI experience between groups, χ^2^(3) = 8.08, *p* = 0.044. Post hoc pairwise comparisons using Dunn’s test with Holm correction indicated that ARP Group 3 had significantly greater CI experience compared to the control group (*padj* = 0.030), while no other group comparisons reached statistical significance.

### 3.4 Hi-MoCA cognitive screening assessment

Overall, the median Hi-MoCA score for all participants was 26 (*IQR*: 4, range: 19–29, *n* = 33). Hi-MoCA scores were compared across Group 1 (25, *IQR*: 2.5, range 22–28, *n* = 8), Group 2 (26, *IQR*:5.25, range 19–29, *n* = 8), Group 3 (25.5, *IQR*: 3.25, range 22–29, *n* = 8) and the control (23, *IQR*: 4, range 14–30, *n* = 9) using the Kruskal-Wallis H test, which revealed no statistically significant differences [χ^2^(3) = 1.61, *p* = 0.66] across participant groups.

### 3.5 eCAEP responses

The eCAEP response data was collected at baseline (T0) and repeated at three follow-up assessments at 10-week intervals (T10-T30). The eCAEP P1-N1-P2 response peak latencies (ms) and amplitudes (μV) were compared pre-post ARP participation as well as from baseline (T0) across assessment intervals (T10-T30), to account for any natural variability and potential ongoing adaptation in auditory performance over time, independent of ARP-related changes.

The Kruskal-Wallis H test was conducted to compare eCAEP P1-N1-P2 response peak latency and amplitude at each data collection time point across apical, medial, and basal electrode contact positions. No significant differences were found for P1 latency [χ^2^(2) = 1.80, *p* = 0.407] or P1 amplitude [χ^2^(2) = 2.24, *p* = 0.327]; N1 latency [χ^2^(2) = 4.77, *p* = 0.092] or N1 amplitude [χ^2^(2) = 3.40, *p* = 0.182]; and P2 latency [χ^2^(2) = 4.07, *p* = 0.131] or amplitude [χ^2^(2) = 0.34, *p* = 0.844]. Therefore, responses from all electrode positions were combined for further analysis.

#### 3.5.1 eCAEP responses: pre- and post-intervention comparison

**FIGURE 2:**
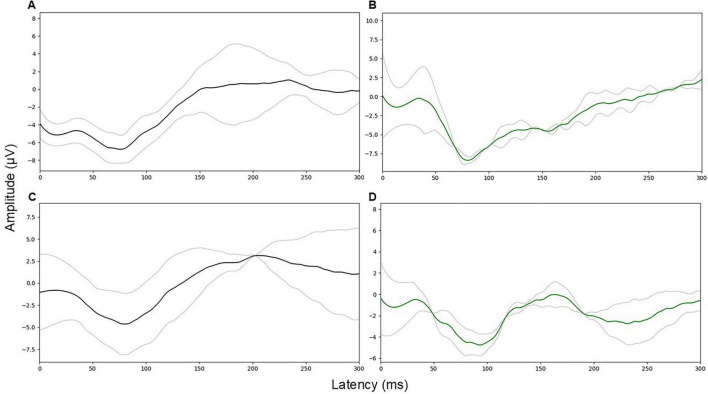
Grand average eCAEP P1-N1-P2 waveforms for pre- (black) and post-intervention (green) conditions. Grey dashed lines represent ± 1 standard deviation. (A,B) The ARP group (pre- and post-intervention), and (C,D) the control group (pre- and post-control period).

**FIGURE 3:**
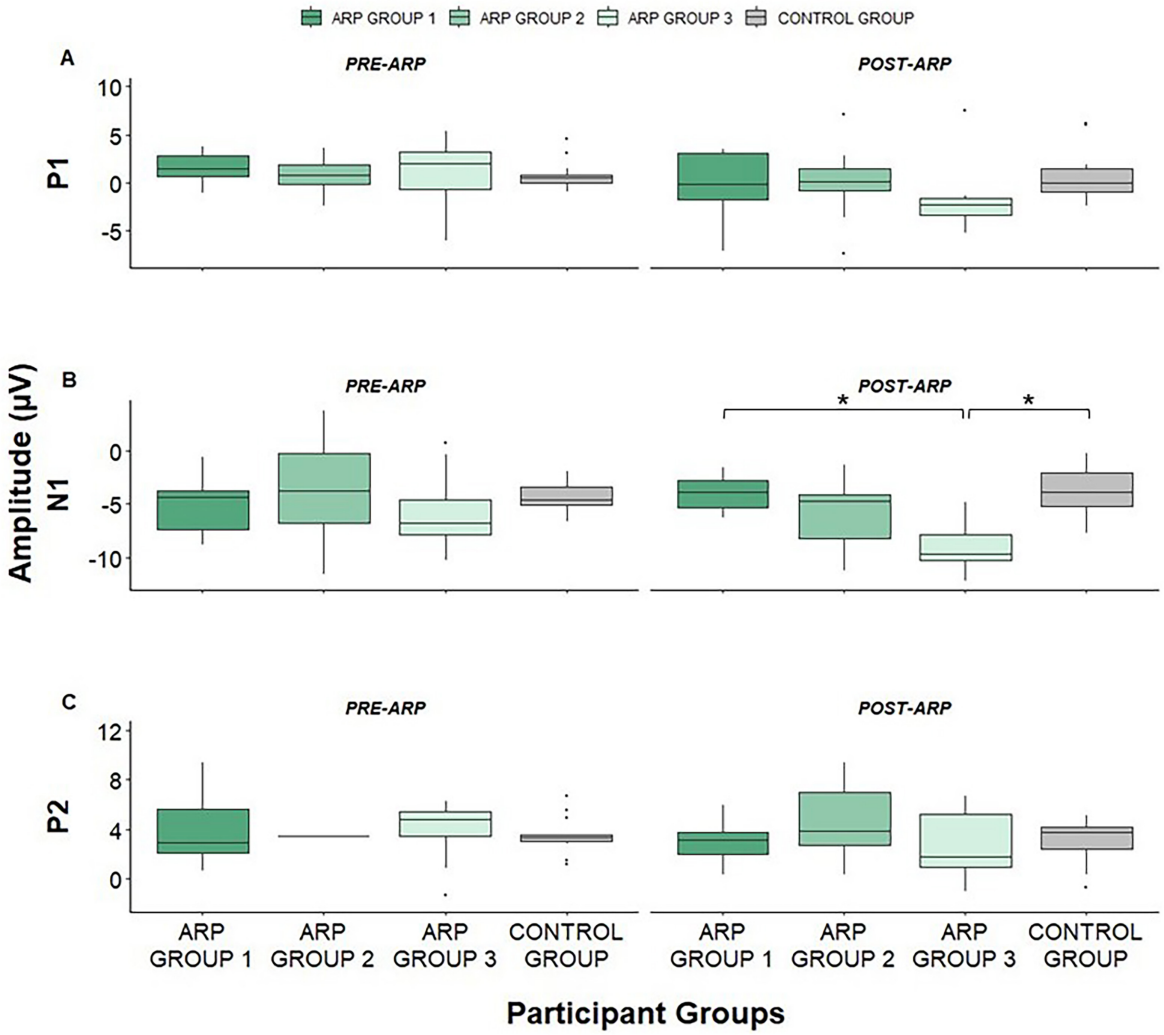
Boxplot depicts median (central line), interquartile range (box) and whiskers extending to show the full range of eCAEP response peak amplitude (μ V) for P1 (A), N1 (B) and P2 (C) between ARP subgroups; Group 1 (*n* = 8), Group 2 (*n* = 8), Group 3 (*n* = 8) and the control (*n* = 11) pre- and post-rehabilitation intervention. Significance following *p*-value adjustment *p < 0.05.

The Wilcoxon rank-sum (Mann-Whitney U) test was used to compare eCAEP P1-N1-P2 response peak latency and amplitude pre- and post-rehabilitation intervention within and between participant groups, with Holm adjustment for multiple comparisons. No significant differences were observed for P1, N1 or P2 response latencies or amplitudes between pre- and post-rehabilitation measurements for both the total ARP and control groups (Figure 2). ARP subgroup analysis showed that the N1 negative deflection amplitude for Group 3 (−9.65μV, *IQR*: 2.38μV) was significantly larger than both Group 1 (−3.85μV, *IQR*: 2.45μV; *U* = 74, *p* = 0.003, *padj* = 0.051) and the control group (−3.85μV, *IQR*: 3.18μV; *U* = 7, *p* = 0.002, *padj* = 0.036) post-rehabilitation intervention (Figure 3B). No significant differences were observed for P1 (Figure 3A) or P2 (Figure 3C) amplitudes between subgroups pre- or post-rehabilitation intervention. No other comparisons of P1, N1, or P2 latency and amplitude between or within ARP sub-groups pre- and post-rehabilitation intervention reached statistical significance, suggesting no objective change in response to the rehabilitation program. Descriptive statistics for eCAEP response peak latency and amplitude by electrode contact position are presented in Table 3.

**TABLE 3:** Combined Aural Rehabilitation Program (ARP) Participant P1-N1-P2 eCAEP Response Peak Latency (ms) and Amplitude (μV): Mean (*SD*), Median (*IQR*), and Range for Apical, Medial, and Basal Electrode Contact Positions Compared Pre- and Post-Intervention.

Electrode Contact	Apical electrode contact		Medial electrode contact		Basal electrode contact	
Position	ARP intervention status		ARP intervention status		ARP intervention status	
	Pre	Post	Pre	Post	Pre	Post
Peak latency (ms)						
P1						
Mean (SD)	43.04 (18.48)	42.53 (15.57)	38.82 (12.43)	34.55 (8.22)	35.73 (9.23)	40.10 (11.21)
Median (IQR)	35.70 (32.10, 49.40)	36.50 (32.90, 44.70)	35.30 (31.30, 43.10)	32.10 (29.80, 41.60)	34.50 (27.40, 43.90)	35.30 (30.60, 47.90)
Range	23.50–89.50	29.80–82.50	25.10–81.70	20.30–44.70	21.90–47.10	27.40–62.00
N1						
Mean (SD)	97.06 (37.15)	87.71 (10.48)	84.56 (14.44)	86.44 (31.36)	83.50 (11.48)	94.08 (36.57)
Median (IQR)	90.30 (78.50, 101.30)	83.20 (81.70, 96.60)	84.00 (77.70, 94.30)	80.50 (69.50, 89.50)	84.80 (78.50, 91.10)	90.30 (84.80, 96.60)
Range	58.10–239.70	64.40–103.70	47.90–106.80	54.10–181.70	62.80–98.20	31.50–180.40
P2						
Mean (SD)	204.97 (26.12)	202.26 (31.05)	213.19 (26.10)	200.49 (30.10)	213.57 (27.21)	215.74 (29.38)
Median (IQR)	202.80 (186.80, 221.70)	202.00 (199.60, 221.70)	207.50 (193.30, 227.20)	202.55 (181.15, 221.25)	213.80 (196.50, 232.70)	213.80 (191.60, 235.00)
Range	148.50–247.60	113.00–238.20	181.50–262.00	139.00–250.80	176.00–258.60	168.20–268.80
Peak amplitude (μV)						
P1						
Mean (SD)	5.09 (20.95)	0.31 (4.16)	1.57 (2.20)	1.02 (4.24)	1.73 (2.04)	−0.42 (2.95)
Median (IQR)	0.45 (−0.60, 1.40)	0.00 (−2.40, 2.30)	1.20 (0.20, 3.60)	0.10 (−2.10, 3.00)	1.20 (0.20, 3.60)	−0.35 (−1.40, 0.90)
Range	−6.00–83.20	−7.10–7.50	−2.40–5.30	−3.70–11.30	−1.10–4.50	−7.40–3.20
N1						
Mean (SD)	−5.72 (4.65)	−5.74 (3.08)	−5.02 (2.77)	−5.26 (3.75)	−2.93 (4.75)	−5.53 (4.17)
Median (IQR)	−5.50 (−7.20, −3.70)	−4.50 (−8.60, −3.20)	−4.50 (−7.30, −3.30)	−4.95 (−7.10, −4.40)	−3.90 (−4.70, −0.40)	−4.50 (−7.50, −1.90)
Range	−16.40–3.70	−10.50 to −2.10	−11.60–0.70	−11.20–3.40	−8.80–6.70	−13.70 to –1.40
P2						
Mean (SD)	3.44 (2.88)	3.34 (2.16)	3.27 (2.05)	2.83 (3.33)	4.69 (1.15)	3.07 (3.09)
Median (IQR)	3.40 (2.50, 4.40)	3.85 (1.80, 5.10)	2.80 (1.70, 5.20)	3.55 (1.75, 3.90)	5.20 (3.10, 5.60)	3.05 (0.30, 4.40)
Range	−2.20–9.40	−0.70–6.60	0.60–6.80	−5.50–7.80	3.00–5.70	−1.00–9.40

#### 3.5.2 eCAEP responses: across ARP timeline

**FIGURE 4:**
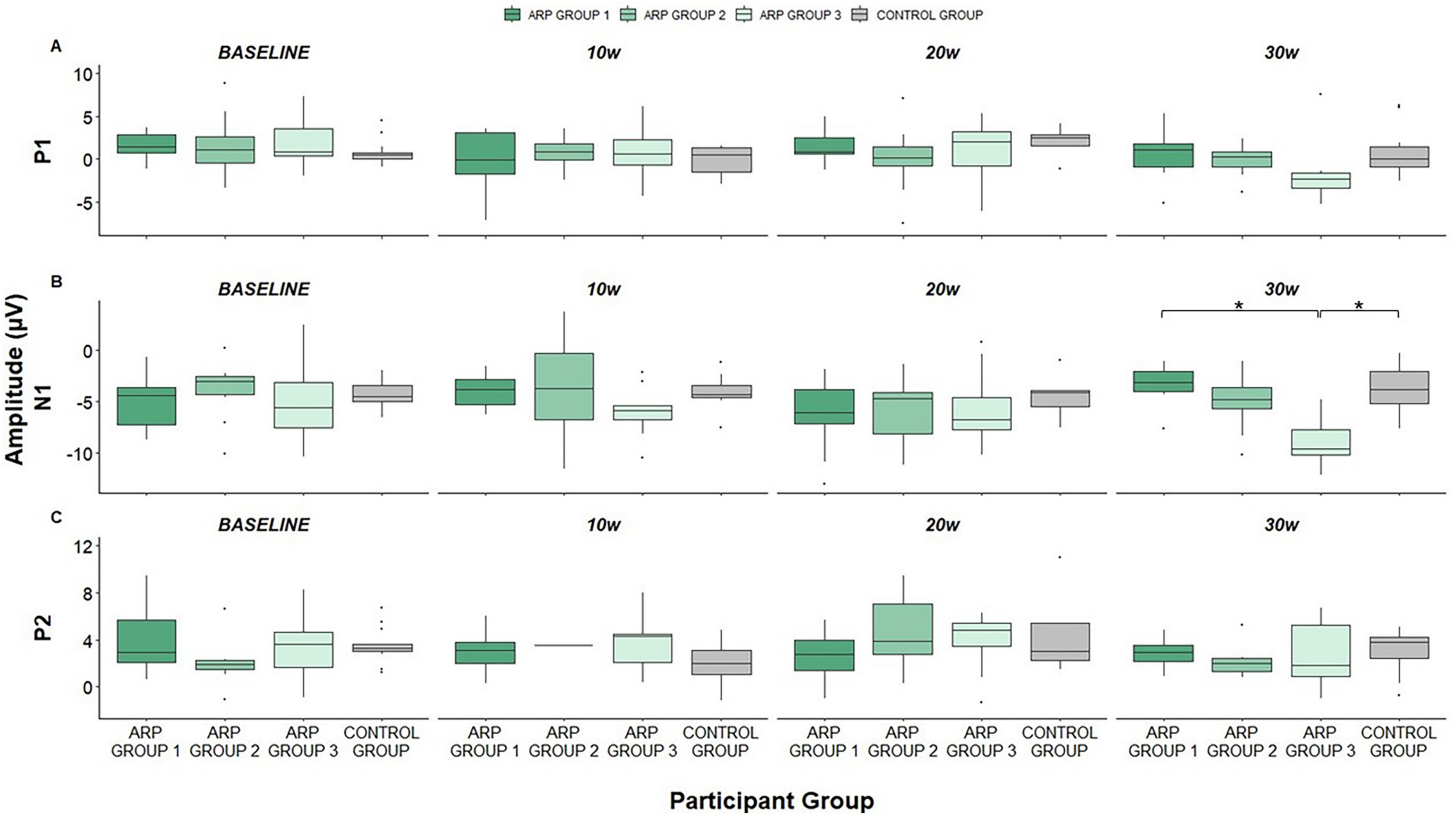
Boxplot depicts median (central line), interquartile range (box) and whiskers extending to show the full range of eCAEP response peak amplitude (μV) for P1 (A), N1 (B) and P2 (C) between ARP subgroups; Group 1 (*n* = 8), Group 2 (*n* = 8), Group 3 (*n* = 8) and the control (*n* = 11) group at baseline (T0) and at each follow-up assessment time point (10w, 20w, 30w). Significance following *p*-value adjustment (*p* < 0.05) is indicated (*).

The Wilcoxon rank-sum (Mann-Whitney U) test was used to compare eCAEP P1-N1-P2 response peak latency and amplitude from baseline (T0) overtime to the final assessment time point (T30) within and between participant groups, with Holm adjustment for multiple comparisons. The N1 negative deflection amplitude was significantly larger for Group 3 (−9.65 μV, *IQR*: 2.38 μV) than both Group 1 (−3.2 μV, *IQR*: 1.95 μV; *U* = 62, *p* = 0.02, padj = 0.048) and the control group (−3.85 μV, *IQR*: 3.18 μV; *U* = 7, *p* = 0.02, padj = 0.048) at 30 weeks (Figure 4B). No significant differences were observed for P1 (Figure 4A) or P2 (Figure 4C) amplitudes between subgroups from baseline (T0) to the final assessment time point (T30). No other comparisons of P1, N1, or P2 latency and amplitude between or within groups overtime (T0-T30) reached statistical significance. Descriptive statistics for eCAEP response peak latencies and amplitudes at baseline and at each follow-up assessment time point (T10, T20, T30) are summarized by electrode contact position in Table 4.

**TABLE 4:** Combined aural rehabilitation program (ARP) participant P1-N1-P2 eCAEP response peak latency (ms) and amplitude (μV): mean (*SD*), median (*IQR*), and range for apical, medial, and Basal electrode contact positions at baseline and each 10-week test interval.

	ARP timeline							
Response	Baseline	10 Weeks	20 Weeks	30 Weeks	Baseline	10 Weeks	20 Weeks	30 Weeks
		Peak latency (ms)				Peak Amplitude (μ V)		
Apical electrode contact								
P1								
Mean (SD)	37.84 (12.91)	45.32 (19.04)	38.82 (15.04)	39.59 (11.78)	6.15 (20.64)	4.25 (19.84)	0.76 (3.39)	0.43 (3.20)
Median (IQR)	34.50 (30.95, 40.40)	47.90 (32.10, 52.60)	34.50 (30.60, 45.50)	36.50 (30.60, 44.70)	0.75 (−0.45, 2.70)	0.40 (−1.90, 1.30)	1.10 (−1.00, 2.50)	0.10 (−1.60, 1.70)
Range	24.30–81.70	14.80–89.50	23.50–82.50	29.00–75.00	−2.00–83.20	−7.10–75.40	−6.00–7.10	−5.20–7.50
N1								
Mean (SD)	91.51 (17.39)	100.96 (40.15)	77.74 (24.92)	88.44 (12.45)	−5.26 (3.45)	7.79 (51.93)	9.23 (59.83)	−5.83 (2.85)
Median (IQR)	87.20 (79.70, 103.30)	94.30 (80.90, 101.30)	80.90 (70.70, 91.15)	83.20 (81.70, 97.80)	−5.20 (−6.75, −3.20)	−4.30 (−6.20, −2.80)	−6.75 (−10.20, −5.10)	−4.90 (−8.15, −3.70)
Range	58.10–139.10	67.50–239.70	9.19–107.60	64.40–110.80	−16.40–0.20	−10.50–215.40	−14.40–198.90	−10.50 to −2.10
P2								
Mean (SD)	202.15 (24.33)	205.93 (23.41)	201.64 (38.67)	205.92 (18.60)	3.84 (2.83)	2.82 (2.22)	3.21 (2.65)	2.97 (1.97)
Median (IQR)	204.35 (190.20, 210.60)	209.85 (185.45, 221.30)	212.25 (186.80, 225.60)	203.95 (199.25, 218.90)	3.20 (2.30, 6.70)	3.50 (1.60, 4.30)	3.90 (0.80, 5.50)	2.85 (1.75, 4.55)
Range	148.50–247.60	170.50–242.10	113.00–241.30	167.40–245.20	−0.90–9.40	−2.20–6.40	−1.30–6.20	−0.70–6.60
Medial electrode contact								
P1								
Mean (SD)	40.48 (12.04)	33.31 (6.47)	34.14 (5.63)	37.85 (13.86)	2.08 (2.59)	1.26 (2.31)	6.23 (19.51)	0.75 (4.52)
Median (IQR)	35.30 (32.90, 44.70)	33.70 (29.80, 38.40)	33.70 (29.82, 37.60)	37.60 (29.00, 43.90)	1.00 (0.50, 3.60)	1.40 (−0.30, 3.00)	0.90 (0.10, 2.60)	−0.70 (−2.40, 1.90)
Range	29.00–81.70	22.70–43.10	25.10–44.70	20.30–73.00	−1.00–8.80	−2.40–6.10	−3.60–70.70	−3.80–11.30
N1								
Mean (SD)	89.21 (25.63)	87.50 (28.65)	77.76 (13.73)	81.03 (17.74)	−4.55 (2.86)	−5.18 (2.54)	−5.92 (3.27)	−4.37 (4.03)
Median (IQR)	89.50 (77.70, 91.90)	84.00 (69.90, 91.10)	79.70 (72.20, 80.90)	78.55 (71.45, 90.30)	−4.40 (−5.90, −2.50)	−5.00 (−6.60, −3.20)	−5.65 (−7.90, −4.20)	−4.55 (−7.75, −1.20)
Range	47.90–180.40	61.20–181.70	44.70–106.80	54.10–118.60	−10.40–0.20	−11.60 to −1.20	−11.20–0.70	−10.10–3.40
P2								
Mean (SD)	213.33 (24.84)	191.54 (26.08)	199.69 (40.40)	216.62 (22.95)	2.77 (2.29)	2.82 (2.37)	3.94 (2.53)	2.26 (3.49)
Median (IQR)	207.10 (194.90, 227.20)	193.30 (169.80, 207.50)	200.40 (169.80, 216.90)	212.20 (203.60, 228.70)	2.50 (1.70, 3.80)	2.60 (1.70, 3.90)	4.00 (2.00, 5.70)	3.50 (1.50, 3.90)
Range	180.20–262.00	139.00–229.50	143.00–280.30	177.60–250.80	−3.00–6.80	−1.20–8.00	0.00–7.80	−5.50–6.70
Basal electrode contact								
P1								
Mean (SD)	41.12 (10.06)	43.45 (15.54)	34.39 (10.83)	38.83 (10.27)	0.81 (2.39)	0.44 (2.87)	1.34 (3.83)	0.18 (3.05)
Median (IQR)	40.40 (33.70, 44.70)	40.40 (31.60, 52.60)	32.50 (25.45, 45.50)	35.70 (32.55, 40.75)	0.65 (−0.55, 1.55)	−0.20 (−1.50, 3.20)	2.65 (0.50, 3.65)	0.05 (−1.20, 1.75)
Range	27.40–59.70	27.40–68.30	20.30–47.90	30.60–62.00	−3.30–6.40	−2.90–3.60	−7.40–4.50	−5.10–5.30
N1								
Mean (SD)	82.72 (10.90)	90.14 (7.03)	88.02 (14.61)	90.10 (37.76)	−3.52 (4.22)	−3.02 (2.37)	5.50 (32.68)	−5.81 (4.83)
Median (IQR)	84.80 (78.50, 91.10)	91.80 (90.30, 92.70)	85.60 (82.50, 89.50)	86.00 (76.20, 99.80)	−4.30 (−4.70, −3.20)	−2.20 (−5.40, −1.60)	−3.35 (−7.50, −1.00)	−4.25 (−5.90, −2.70)
Range	62.80–98.20	78.50–97.40	73.00–125.50	31.50–180.40	−8.80–6.70	−5.60 to −0.30	−13.70–97.80	−16.40 to −1.10
P2								
Mean (SD)	217.23 (34.98)	190.15 (36.10)	221.96 (18.59)	223.61 (28.38)	3.33 (2.12)	3.20 (2.39)	4.35 (3.92)	3.50 (4.40)
Median (IQR)	217.35 (189.15, 245.65)	179.90 (164.25, 216.05)	227.95 (209.10, 234.25)	218.50 (202.40, 244.85)	3.05 (2.05, 5.30)	3.25 (1.45, 4.95)	3.00 (1.45, 7.30)	2.60 (1.05, 4.15)
Range	163.50–272.00	160.30–240.50	191.00–242.10	188.60–268.80	−1.10–5.70	0.30–6.00	0.30–11.00	−1.00–13.40

#### 3.5.3 eCAEP response: linear mixed model analysis

**FIGURE 5:**
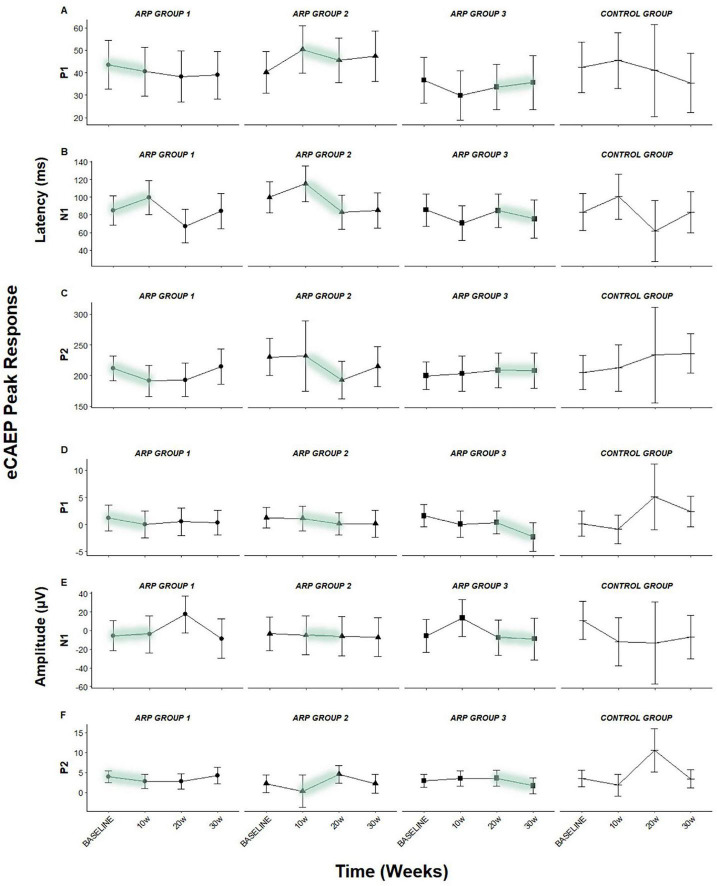
Line graph depicts eCAEP response P1 (A), N1 (B) and P2 (C) latency (ms) and P1 (D), N1 (E) and P2 (F) amplitude (μV) overtime from baseline (T0) to follow-up assessment time points (10W, 20W, 30W). Error bars indicate the estimated marginal means and highlighted line regions illustrate ARP intervention periods for each subgroup.

A linear mixed model was performed to analyze the effect of time (baseline, T10, T20, T30), ARP sub-group (Group 1, Group 2, Group 3, and control), test electrode contact position (apical, medial, basal) and Hi-MoCA score on eCAEP P1-N1-P2 response peak amplitude and latency. Random intercepts for participants (ID) were included to account for individual variability. Linear mixed models were conducted for eCAEP response amplitude and latency for each P1, N1 and P2 waveform component; estimated marginal means are presented across time for each ARP sub-group (Figure 5).

No significant main or interaction effects were found for P1 amplitude or latency. The model revealed a significant main effect of time on N1 latency [*F*(3, 131.224) = 3.66, *p* = 0.0142]. Pairwise contrasts using estimated marginal means revealed a significant difference between T10 and T20 (Estimate = −22.38, *SE* = 7.16, *df* = 130, *t* = 3.125, *p* = 0.0131), suggesting a notable decrease in latency between these time points. However, no significant effects or interactions were observed for N1 latency involving ARP sub-group, test electrode contact position, or Hi-MoCA score, and no significant effects were detected for N1 amplitude.

The LMM showed that increased P2 latency is significantly associated with the basal electrode contact position (Estimate = 21.36, *SE* = 9.49, *df* = 97.63, *t* = 2.25, *p* = 0.027), however, the effect of electrode contact position was not statistically significant [*F*(3, 95.44) = 2.68, *p* = 0.074]. Although emmeans results suggest that the basal electrode generally leads to longer latencies than the apical electrode, pairwise comparisons with *p*-value adjustment indicate that the difference between apical and basal P2 latency is not statistically significant (Estimate = -21.36, *SE* = 9.61, *df* = 97.3, *t* = -2.22, *p* = 0.085). No other significant effects were detected for P2 latency.

The model revealed P2 amplitude is significantly associated with time [*F*(3, 98.975) = 3.41, *p* = 0.0204. Pairwise contrasts demonstrated a significant increase from T0 to T20 (Estimate = -2.24, *SE* = 0.92, *df* = 94.5, *t* = -2.43, *p* = 0.0344) and T10 to T20 (Estimate = -3.25, *SE* = 1.05, *df* = 99.3, *t* = -3.09, *p* = 0.0154), followed by a significant decrease from T20 to T30 (Estimate = 2.49, *SE* = 0.97, *df* = 95.3, *t* = 2.57, *p* = 0.0344). Estimated marginal means indicated P2 amplitude was greatest at T20 (5.37 μV, *SE* = 0.819 μV, *df* = 83.2, [3.74 μV, 7.00 μV]), before declining at T30 (2.88 μV, *SE* = 0.543 μV, *df* = 71.1, [1.80 μV, 3.96 μV]). No other main effects or interactions reached statistical significance.

### 3.6 Speech perception

Speech perception testing was conducted using the CNC speech test at baseline (T0) and repeated at three follow-up assessments at 10-week intervals (T10-T30). CNC phoneme and word scores (%) were compared pre-post rehabilitation intervention as well as from baseline (T0) to the final assessment interval (T30), to account for any natural variability and potential ongoing adaptation in auditory performance over time, independent of ARP-related changes. Descriptive statistics for CNC phoneme and word scores (%) in both implant-alone and binaural listening conditions are presented across ARP subgroups over time (T0-T30) in Table 5.

**TABLE 5:** Aural rehabilitation program (ARP) speech in quiet (CNC) speech perception phoneme and word scores (%): mean (SD), median (IQR), and range at baseline and each 10-week test interval.

ARP timeline									
Group	CNC score (%)	Baseline (T0)		10 weeks (T10)		20 weeks (T20)		30 weeks (T30)	
		Phoneme	Word	Phoneme	Word	Phoneme	Word	Phoneme	Word
**ARP group 1**	**Implant-alone**								
	Mean (SD)	37 (14)	19 (13)	60 (13)	32 (14)	61 (14)	33 (18)	62 (18)	36 (20)
	Median (IQR)	40 (29–47)	24 (12–28)	61 (46–72)	33 (28–40)	64 (56–71)	38 (20–46)	67 (49–74)	42 (22–50)
	Range	12–53	0–32	40–76	8–48	36–76	8–48	36–77	8–52
	**Binaural**								
	Mean (SD)	63 (10)	44 (15)	67 (19)	37 (24)	72 (13)	39 (20)	68 (31)	51 (32)
	Median (IQR)	61 (58–68)	48 (32–48)	68 (58–85)	29 (24–48)	73 (63–81)	48 (29–50)	76 (44–91)	59 (27–76)
	Range	50–83	26–64	33–92	12–80	52–89	9–52	25–93	8–80
**ARP Group 2**	**Implant-alone**								
	Mean (SD)	62 (12)	37 (17)	50 (21)	29 (19)	63 (16)	35 (20)	75 (17)	51 (26)
	Median (IQR)	64 (50–73)	32 (24–56)	42 (33–75)	28 (16–52)	67 (44–75)	36 (12–56)	74 (64–86)	44 (36–66)
	Range	50–73	24–56	26–76	4–56	40–83	8–56	55–96	28–88
	**Binaural**								
	Mean (SD)	75 (18)	56 (23)	79 (15)	58 (26)	89 (5)	70 (12)	79 (18)	56 (32)
	Median (IQR)	75 (62–87)	56 (40–72)	80 (61–93)	52 (32–84)	88 (84–93)	70 (60–80)	83 (65–92)	60 (30–82)
	Range	62–87	40–72	60–97	24–96	83–95	56–84	55–93	16–88
**ARP group 3**	**Implant-alone**								
	Mean (SD)	56 (16)	29 (18)	50 (9)	16 (21)	42 (19)	21 (16)	64 (12)	35 (16)
	Median (IQR)	60 (39–70)	40 (8–40)	50 (41–58)	8 (0–40)	48 (28–57)	22 (10–32)	57 (57–67)	32 (24–40)
	Range	39–70	8–40	41–58	0–40	16–58	0–40	57–84	20–60
	**Binaural**								
	Mean (SD)	53 (12)	27 (15)	51 (13)	21 (13)	67 (11)	46 (16)	76 (5)	52 (8)
	Median (IQR)	60 (40–60)	30 (10–40)	51 (41–60)	21 (12–30)	61 (61–80)	42 (32–64)	76 (75–77)	52 (44–56)
	Range	40–60	10–40	41–60	12–30	61–80	32–64	70–84	44–64
**Control group**	**Implant-alone**								
	Mean (SD)	34 (19)	17 (20)					39 (25)	16 (13)
	Median (IQR)	31 (20–50)	12 (2–25)					44 (35–53)	16 (8–20)
	Range	0–63	0–58					0–73	0–40
	**Binaural**								
	Mean (SD)	49 (29)	20 (24)					55 (25)	32 (19)
	Median (IQR)	56 (24–75)	12 (4–36)					63 (47–72)	33 (20–48)
	Range	0–79	0–55					0–73	0–52

#### 3.6.1 Speech perception: pre- and post-intervention comparison

**FIGURE 6:**
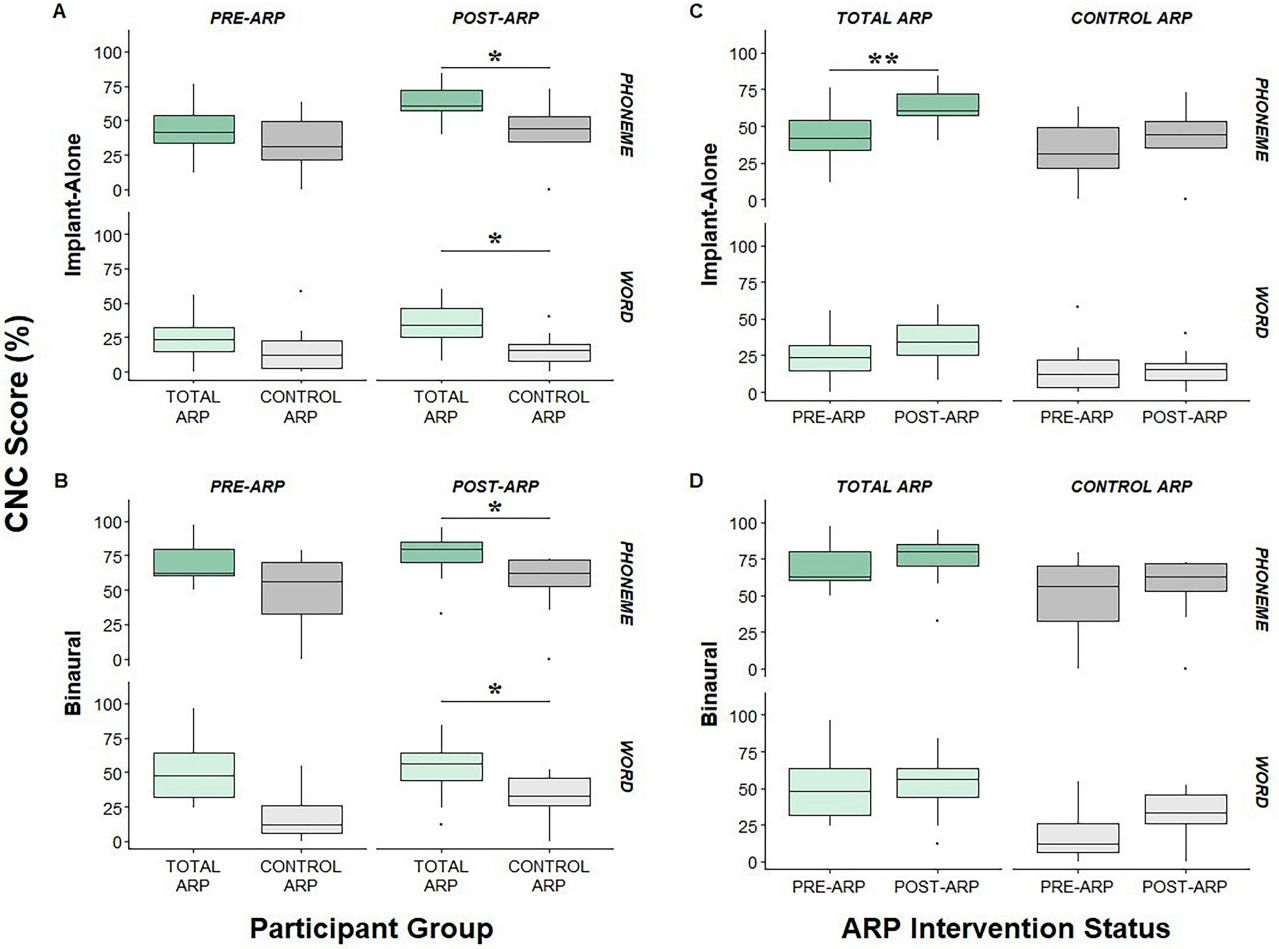
Boxplot depicts median (central line), interquartile range (box), and whiskers representing the full range of CNC phoneme (top row) and word (bottom row) scores (%) for inter-group **(A,B)** and intra-group **(C,D)** comparisons pre- and post-rehabilitation intervention in the implant-alone **(A,C)** and binaural conditions **(B,D)**. Significance following *p*-value adjustment is denoted as *p < 0.05, **p < 0.005.

Speech perception scores were analyzed pre- and post-rehabilitation for the total ARP group (*n* = 24) and control group (*n* = 11) to evaluate inter- (Figures 6A,B) and intra-group (Figures 6C,D) changes following the rehabilitation intervention.

Inter-group comparisons confirmed that pre-rehabilitation phoneme and word scores did not differ significantly between the total ARP and control groups in implant-alone (Figure 6A) or binaural conditions (Figure 6B). Post-rehabilitation intervention, the ARP group achieved a significantly higher phoneme (61%, *IQR* 15%) and word (34%, *IQR* 21%) score than the control group (phoneme score: *U* = 136 *p* = 0.0128, *padj* = 0.0256; word score: *U* = 130, *p* = 0.012, *padj* = 0.023) in the implant-alone condition (Figure 6A). Furthermore, the ARP group achieved a significantly higher phoneme (80%, *IQR* 14.8%) and word (56%, *IQR* 20%) score than the control group (phoneme score: *U* = 117, *p* = 0.0133, *padj* = 0.0266; word score: *U* = 104, *p* = 0.038 *padj* = 0.05) in the binaural condition (Figure 6B).

Following these findings, intra-group comparisons revealed that the total ARP group demonstrated a significant improvement in phoneme scores from pre- (42%, 20.5%) to post-rehabilitation (61%, 15%) in the implant-alone condition (*U* = 5.5, *p* = 0.00053, *padj* = 0.0011; Figure 6C). Additionally, while the ARP group showed an increase in the word score between pre- (24%, 17%) and post-rehabilitation (34%, 21%), this was not statistically significant (Figure 6C). The control group did not demonstrate a significant change for the phoneme or word scores. No significant change in phoneme or word scores was observed pre- to post-rehabilitation intervention for the in the binaural condition for both the total ARP and control groups (Figure 6D).

#### 3.6.2 Speech perception: ARP sub-group pre- and post-intervention comparison

**FIGURE 7:**
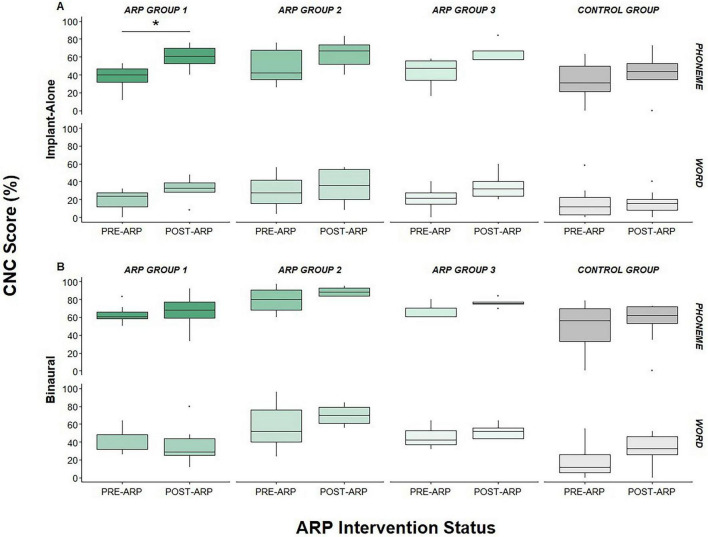
Boxplot depicts median (central line), interquartile range (box) and whiskers representing the full range of CNC phoneme (top row) and word (bottom row) scores (%) for intra-group comparisons pre- and post-rehabilitation intervention within each ARP sub-group; Group 1 (*n* = 8), Group 2 (*n* = 8), Group 3 (*n* = 7) and the control group (*n* = 11). Testing was conducted in implant-alone **(A)** and binaural **(B)** conditions. Significance following *p*-value adjustment (*p* < 0.05) is indicated (*).

Speech perception scores were analyzed pre- and post-rehabilitation among participant subgroups; Group 1 (*n* = 8), Group 2 (*n* = 8), Group 3 (*n* = 7), and the control group (*n* = 11) to evaluate inter- and intra-group (Figure 7) changes following the rehabilitation intervention.

Inter-group comparisons confirmed that pre-rehabilitation phoneme and word scores did not differ significantly between the ARP subgroups and control groups in implant-alone or binaural conditions. In contrast, intra-group comparisons revealed that only ARP Group 1 demonstrated a marginally significant improvement in phoneme score from pre-rehabilitation (40%, *IQR* 15%) to post-rehabilitation (61%, *IQR* 17%) in the implant-alone condition (*U* = 0, *padj* = 0.0524; Figure 7A). This improvement did not reach statistical significance for the word score in the implant-alone condition (Figure 7A) or the phoneme and word scores in the binaural condition (Figure 7B). Phoneme and word scores in ARP Groups 2 and 3 showed an increasing trend post-rehabilitation intervention in both implant-alone (Figure 7A) and binaural (Figure 7B) conditions, however, these changes were not statistically significant. The control group exhibited no significant score changes in either condition (Figure 7).

Following these findings, exploratory Spearman’s rank-order correlations were conducted to examine the relationship between total duration of CI experience and speech perception performance in both the implant-alone and binaural conditions (phoneme and word scores) for each ARP sub-group at pre- and post-rehabilitation test intervals. Across all analyses, no significant (all *p* > 0.05) correlations were observed, and results remained non-significant following correction for multiple comparisons. Overall, total duration of CI experience was not significantly associated with speech perception scores pre- or post-ARP intervention, suggesting that changes in performance were independent of duration of CI experience within the present cohort.

#### 3.6.3 Speech perception: across ARP timeline

**FIGURE 8:**
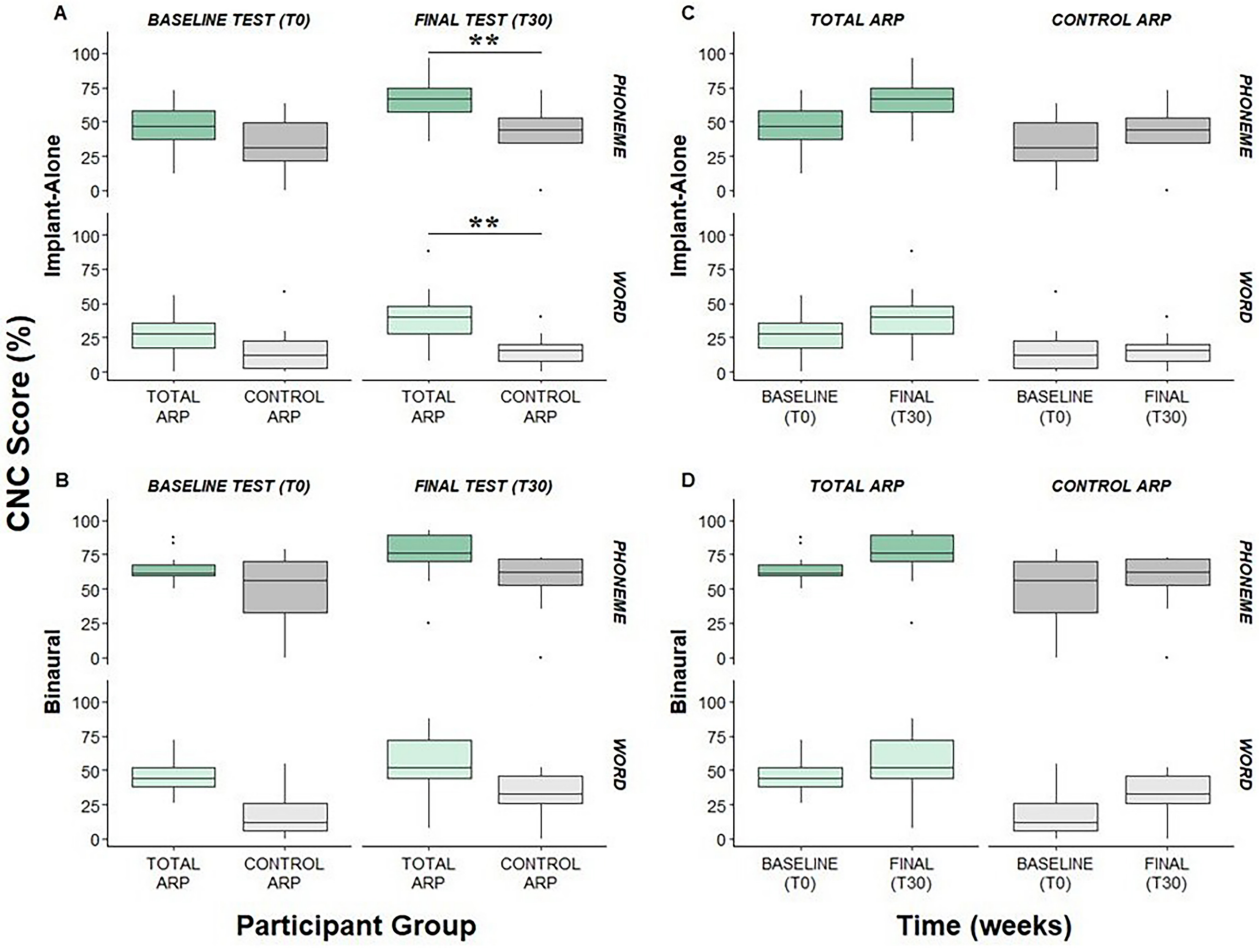
Boxplot depicts median (central line), interquartile range (box) and whiskers representing the full range of CNC phoneme (top row) and word (bottom row) scores (%) for inter-group **(A,B)** and intra-group **(C,D)** comparisons at baseline (T0) and the final assessment time point (T30) in the implant-alone **(A,C)** and binaural conditions **(B,D)**. Significance following *p*-value adjustment is denoted as **p < 0.05.

Speech perception scores were analyzed at baseline (T0) and the final assessment time point (T30) for the ARP group (*n* = 24) and control group (*n* = 11) to evaluate inter- (Figures 8A,B) and intra-group (Figures 8C,D) changes overtime.

Inter-group comparisons confirmed that phoneme and word scores did not differ significantly between the total ARP and control groups at baseline in implant-alone (Figure 8A) or binaural conditions (Figure 8B). After 30 weeks, the total ARP group showed a significantly higher phoneme (67%, *IQR* 18%) and word (40%, *IQR* 20%) score than the control group (phoneme: *U* = 99, *p* = 0.0075, *padj* = 0.022, word: *U* = 102, *p* = 0.004 *padj* = 0.018) in the implant-alone condition (Figure 8A). Similarly, the total ARP group achieved a higher phoneme score (76%, *IQR* 19%) and word score (52%, *IQR* 28%) compared the control group in the binaural condition, however, this change was not statistically significant following *p*-value adjustment (phoneme: *U* = 84, *p* = 0.022 *padj* = 0.089, word: *U* = 81, *p* = 0.038 *padj* = 0.11) in the binaural condition (Figure 8B).

Intra-group comparisons over time revealed that the total ARP group demonstrated an increase in phoneme score from baseline (47%, *IQR*: 20.8%) to 30 weeks (67%, IQR: 18%) in the implant-alone condition (Figure 8C) and from baseline (61%, *IQR*: 8%) to 30 weeks (76%, *IQR*: 19%) in the binaural condition (Figure 8D), though these improvements were not statistically significant. Similarly, word scores increased in both conditions: implant-alone (baseline: 28%, *IQR*: 18%; 30 weeks: 40%, *IQR*: 20%; Figure 8C) and binaural (baseline: 44%, *IQR*: 14%; 30 weeks: 52%, *IQR*: 18%; Figure 8D), however, no significant changes were observed. The control group showed no significant change in either phoneme or word scores in the implant-alone (Figure 8C) or binaural (Figure 8D) conditions. Overall, intra-group comparisons from baseline to the 30-week test interval yielded non-significant differences within both ARP and control groups.

#### 3.6.4 Speech perception: linear mixed model analysis

**FIGURE 9:**
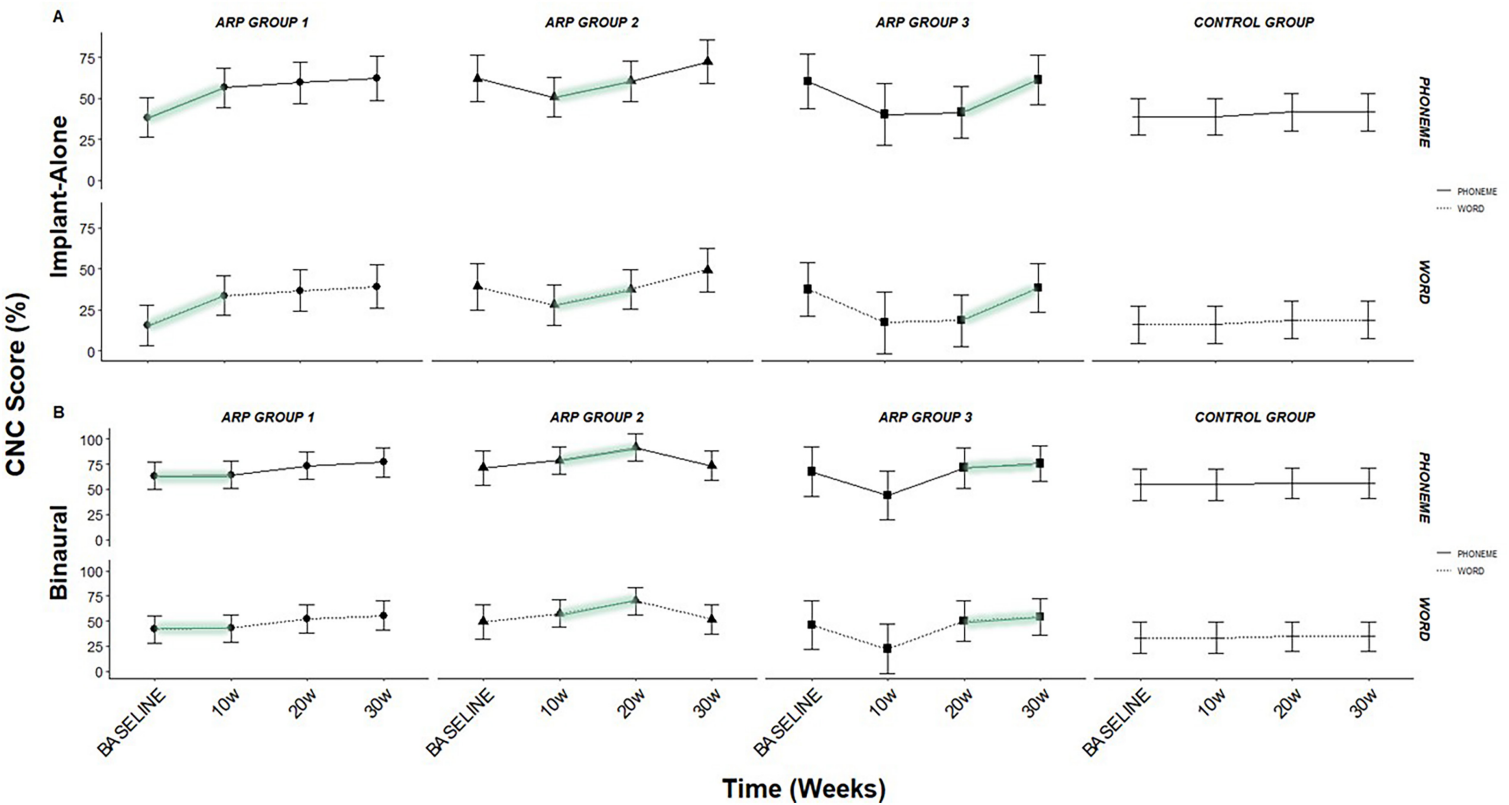
Line graph depicts CNC phoneme and word scores overtime from baseline (T0) across follow-up assessment time points (10w, 20w, 30w). Error bars indicate the estimated marginal means for the implant-alone **(A)** and binaural **(B)** conditions and highlighted line regions illustrate ARP intervention periods for each subgroup.

Linear mixed model analysis was used to examine the effects of time (baseline, T10, T20, T30), ARP subgroups (Group 1, Group 2, Group 3, and control), and Hi-MoCA scores on CNC phoneme and word scores. The model included random intercepts for participants (ID) to account for individual variability. Linear mixed models were conducted for CNC scores in both the implant-alone and binaural test conditions; estimated marginal means are presented across time for each ARP sub-group (Figure 9).

A significant main effect of time [*F*(3, 39.35) = 4.26, *p* = 0.011] was found for CNC phoneme score in the implant-alone condition, with significant interactions between time and rehabilitation group [*F*(7, 39.26) = 3.71, *p* = 0.004; Figure 9A]. Estimated marginal means pairwise contrasts revealed Group 1 showed a significant increase over time from baseline to T10 (Estimate = 21.297, *SE* = 6.08, *df* = 38, *t* = -3.502, *p* = 0.0273), T20 (Estimate = -23.509, *SE* = 6.42, *df* = 38.8, *t* = -3.660, *p* = 0.0227) and T30 (Estimate = -25.398, *SE* = 7.44, *df* = 39.3, *t* = -3.412, *p* = 0.0274; Figure 9A). Hi-MoCA score did not significantly influence phoneme scores, nor did it interact significantly with rehabilitation group. No significant main effects or interactions were found for CNC word scores in the implant-alone or binaural conditions.

## 4 Discussion

This study evaluated a structured ARP intervention in long-term adult CI users with poor baseline performance by assessing pre- to post-rehabilitation changes in neurophysiological responses using eCAEP measures alongside behavioral speech perception scores. Participants completed a 10-week intensive ARP within a 30-week repeated-measures design, with assessments conducted at baseline (T0) and at 10-week intervals (T10, T20, T30). Changes in eCAEP response amplitudes (μV) and latencies (ms) as well as CNC phoneme and word recognition scores (%), were analyzed pre- and post-intervention to evaluate ARP effects and across the 30-week ARP timeline to account for natural variability and auditory adaptation independent of the intervention. This study hypothesized that combining electrophysiological measures with traditional behavioral assessments would offer objective insights into the neuroplastic effects of auditory training in adult CI users, to more accurately assess the benefits of aural rehabilitation and its role in optimizing post-implantation speech perception outcomes.

### 4.1 Outcomes observed pre- and post-ARP intervention

The present findings did not support the hypothesis, as no significant changes were observed in eCAEP response amplitudes or latencies when comparing pre- and post-rehabilitation intervention. Nevertheless, improvements in speech perception in quiet, as measured by CNC phoneme and word scores in the present study, provide encouraging evidence for the benefits of auditory training and rehabilitation. Specifically, post-rehabilitation comparisons revealed that the ARP intervention group achieved significantly higher phoneme and word scores than the control group in both the implant-alone and binaural conditions. Following rehabilitation, median phoneme scores increased from 62.5 to 80% binaurally and from 42 to 61% with the implant alone, demonstrating the benefits of the ARP in enhancing speech perception. While only one ARP subgroup showed significant improvement, all demonstrated overall gains in speech perception scores. This may reflect baseline variability in the context of CI experience, as training benefits are often greater in those with less than 1 year of experience (Fu et al., 2005). Given that all participants had over 12 months of CI use, this may explain the observed outcomes. Based on prior literature indicating that shorter CI experience ( < 3 years) may be associated with greater functional improvement (Moberly et al., 2018), exploratory analyses were conducted to compare outcomes by total CI experience. No significant association or difference was found in the magnitude of speech perception improvements following ARP. However, the significant improvement in the total ARP group, with no change in controls, further supports the benefit of the program.

These findings align with several prior studies that have examined the effects of short-term auditory training on speech perception in adults with CIs (Fu and Galvin, 2007; Stacey et al., 2010). Fu et al. (2005) demonstrated improved speech perception in quiet following a four-week training program, highlighting the effects of short-term rehabilitation. However, a subsequent study by the same authors highlighted the impact of baseline performance variability on outcomes and recommended within-subject control designs to better assess training efficacy (Fu and Galvin, 2008).

### 4.2 Outcomes across ARP timeline

In line with these recommendations, the current study incorporated inter- and intra-participant comparisons over a 30-week period to assess baseline performance and variability in changes over time. Analysis of eCAEP response amplitudes and latencies across assessment intervals revealed significant temporal changes, with N1 latency decreasing from 10 to 20 weeks, and P2 amplitude increasing from baseline to 20 weeks before decreasing at 30 weeks. However, these changes were not influenced by the rehabilitation group, suggesting that this may reflect natural neuroplastic processes rather than intervention effects. These results are consistent with previous studies on auditory processing and cortical plasticity post-implantation, supporting the natural course of auditory neural adaptation (Moore and Shannon, 2009; Sandmann et al., 2015).

Additionally, comparisons of speech perception scores across assessment intervals showed no significant differences between the ARP and control groups at baseline in both the implant-alone and binaural conditions. At the 30-week interval, the ARP group achieved significantly higher phoneme and word scores than the control group in the implant-alone condition. However, within-group comparisons from T0 to T30 did not reach statistical significance. Therefore, these findings across assessment intervals should be interpreted cautiously and are best considered as supportive of between-group differences over time rather than definitive evidence of sustained intervention-related improvement. Together with the pre-post ARP findings, these results suggest potential functional benefit of aural rehabilitation, while highlighting variability in longer-term outcomes.

### 4.3 Objective verification of aural rehabilitation benefit using eCAEP responses

The present results align with previous research on short-term computer-based auditory psychophysical training in adult CI users, which investigated the impact on speech recognition and its reflection in CAEP responses (Barlow et al., 2016). Barlow et al. (2016) found that auditory training was associated with small but significant improvements in speech perception and spectral discrimination, although CAEP changes did not correlate with behavioral measures, a finding consistent with the current study. However, while no correlation between CAEP and behavioral measures was observed in both studies, Barlow’s study showed a significant increase in CAEP N1P2 amplitude post-training, using a speech stimulus. In contrast, the present study employed electrical burst stimuli for electrically-evoked CAEP response recording, which may explain differences in findings. Furthermore, although an increase in N1 amplitude was observed in Group 3 following ARP intervention, consistent with Barlow et al. (2016), this finding was not consistent across groups in the present study. While it may indicate a potential neural correlate of training in a subset of participants, it should be interpreted cautiously given the small sample size and inter-individual variability in responses.

Nevertheless, learning-related cortical plasticity has been found after discrimination training using tones (Näätänen et al., 1993) and synthetic speech stimuli (Kraus et al., 1995; Tremblay et al., 2001, 2009) providing further evidence that auditory training can modify neurophysiological responses in the central auditory system, with increased neural activity correlating with perceptual improvements (Atienza et al., 2002; Song et al., 2012; Tremblay et al., 2001, 2009; Wisniewski et al., 2020). However, individual variability in response to auditory training exists, whereby some individuals exhibit delayed or absent improvements (Anderson and Kraus, 2013; Tremblay, 2007; Tremblay et al., 1998). Additionally, the temporal relationship between these changes and perceptual performance remains unclear (Atienza et al., 2002). The limited understanding of neural plasticity time courses following auditory training, particularly in post-lingually deafened adults, may explain the absence of CAEP changes in the present study. The relatively short rehabilitation period may have been insufficient for significant cortical reorganization, as this process requires sustained auditory input over time (Kral and Sharma, 2012; Sharma et al., 2005). This further raises the possibility that electrically-evoked CAEPs using tone burst stimuli may lack sufficient sensitivity to detect subtle neural plasticity associated with speech processing. In contrast, speech-evoked CAEPs may provide a more representative measure of cortical reorganization occurring with rehabilitation in adult CI users (Friesen and Tremblay, 2006; Martin et al., 2008). Additionally, neuroplasticity in adult CI users may be less pronounced compared to that observed in pediatric populations following CI rehabilitation (Gilley et al., 2008; Kral and Sharma, 2012). Moreover, cortical changes in adult CI users may be more pronounced within the first 12 months post-implantation or require more intensive stimulation to elicit measurable responses (Abbas and Brown, 2015; Lightfoot, 2016).

### 4.4 Potential factors contributing to variability in aural rehabilitation benefit

Individual variability in auditory training outcomes may be influenced by cognitive, motivational, or other external factors (Akeroyd, 2008; Humes and Dubno, 2009; Humes et al., 2016). Consequently, baseline cognitive abilities and the interaction between auditory perception and cognition are of growing importance, especially in populations with age-related hearing loss. In light of the potential link between hearing loss, aging, and cognitive decline (Humes et al., 2010; Humes et al., 2013; Peelle and Wingfield, 2016; Peelle et al., 2011; Slade et al., 2020; Wayne and Johnsrude, 2015), and the older age of the present cohort, the potential impact of participant age and cognitive status should be considered.

Age-related alterations in auditory processing and cortical activation have been observed in individuals with hearing loss, such as larger P2 amplitudes (Bertoli et al., 2011; Campbell and Sharma, 2013), as well as delayed N1 and P2 latencies, which may reflect increased effortful listening (Bertoli et al., 2011; Tremblay et al., 2003) or inefficient cortical processing due to the demands of interpreting degraded auditory input (Campbell and Sharma, 2013; Peelle and Wingfield, 2016). In the present study, however, no significant differences were found in participants’ age at program enrolment or age at implantation, and no correlation was observed between Hi-MoCA score and eCAEP response latency or amplitude.

To further assess potential cognitive influences, the Hi-MoCA screening was incorporated to evaluate the cognitive profile of the cohort. While no significant differences were observed across participant groups, the Hi-MoCA scores ranged from 19 to 29, with a median score of 26, indicating that while most participants passed the screening, some displayed mild cognitive impairment. Despite this, no interaction was found between eCAEP response amplitudes or latencies and participants’ Hi-MoCA scores. This suggests that cognitive decline, if present, may not have influenced neurophysiological measures during the study period. However, it is important to consider that the short duration of the study may have been insufficient to detect changes in cognitive function, which may occur over a longer period post-implantation (Völter et al., 2022a). Additionally, there is evidence to suggest that cognitive status in adults is a predominant factor accounting for individual differences in aided speech-understanding performance (Akeroyd, 2008; Beyea et al., 2016; Humes et al., 2010; Humes et al., 2013). In light of this, the present study examined the correlation between participant Hi-MoCA and speech perception scores, but found no significant interaction, suggesting that cognitive status did not have a meaningful impact on speech improvement in this context. However, this discrepancy may be due to prior studies demonstrating this is most apparent for speech understanding in competing speech or noise stimuli (Akeroyd, 2008; Heinrich et al., 2016; Humes and Dubno, 2009; Humes et al., 2016; Moradi et al., 2014), whereas the present study assessed speech perception in quiet.

While electrophysiological measures did not capture significant neuroplastic changes in this study, the improvements in speech perception observed in a subset of long-term users with low baseline performance suggest that structured aural rehabilitation may offer functional benefits for some individuals post-CI. However, these gains were relatively small and not consistently statistically significant. Therefore, the absence of pre-post changes in eCAEP measures may reflect the limited magnitude of behavioral change observed. As such, the present results do not provide sufficient evidence to discount the utility of eCAEP measures as an objective marker of aural rehabilitation-induced neuroplasticity in long-term CI users, particularly in the short term.

### 4.5 Limitations

This study may be limited by using electrical burst stimuli to evoke CAEP responses. Although the P1-N1-P2 complex is a well-established marker of auditory cortical activation in both adults and children (Chang et al., 2012; Korczak et al., 2005; Lightfoot, 2016; Visram et al., 2023), this approach may have reduced sensitivity in detecting subtle neuroplastic changes in adult CI users. The use of speech-evoked CAEP measures may enhance the detection of these changes in future research (Martin et al., 2008; Tong et al., 2009). Furthermore, these findings could be further extended in future studies using larger samples and parallel-group designs. Although the staggered design supported within-subject comparisons, it may have reduced statistical power and introduced variability in follow-up durations across groups.

In addition, as this study focused on an intensive 10-week clinician-led, face-to-face structured program, data on participants’ self-directed home-based rehabilitation were not collected. Accordingly, potential variability in unsupervised auditory training should be considered when interpreting the present findings. Future studies would benefit from incorporating training logs or objective digital tracking to more accurately quantify the duration and frequency of auditory training and to further understand its relationship with rehabilitation outcomes.

These findings suggest that while auditory training remains beneficial for improving behavioral outcomes, the use of eCAEP measures may not serve as a reliable marker of aural rehabilitation-induced neuroplasticity in the short term in this population of long-term CI users. Further investigation is warranted to explore alternative neural markers, particularly for long-term users who may not exhibit substantial cortical plasticity changes. Future research should explore the interaction between neural and behavioral plasticity, assessing whether modifications in training intensity, duration, or stimuli complexity could yield measurable CAEP adaptations alongside speech perception gains.

## 5 Conclusion

This study evaluated a structured aural rehabilitation program in long-term adult CI users with poor baseline performance by examining pre- to post-intervention changes in neurophysiological responses using eCAEP measures as well as behavioral speech perception scores. While eCAEP responses did not demonstrate significant measurable changes following rehabilitation, the observed improvements in speech perception outcomes support the role of auditory training in adult long-term CI users with poor baseline performance. Overall, these findings highlight the clinical value of aural rehabilitation and may inform future research and clinical practice to support the integration of structured rehabilitation programs as an avenue for improving long-term outcomes post-implantation, particularly in users with poor performance.

## Data Availability

The original contributions presented in the study are included in the article/supplementary material, further inquiries can be directed to the corresponding author.
